# Evolution of long COVID over two years in hospitalised and non-hospitalised survivors in Bangladesh: a longitudinal cohort study

**DOI:** 10.7189/jogh.15.04075

**Published:** 2025-03-14

**Authors:** Farzana Afroze, Shohael Mahmud Arafat, Chowdhury Meshkat Ahmed, Baharul Alam, Sayera Banu, Md Zahidul Islam, Mustafa Mahfuz, Firdausi Qadri, Taufiqur Rahman Bhuiyan, Irin Parvin, Mst Mahmuda Ackhter, Farhana Islam, Monjeline Sultana, Eva Sultana, Mohammad Ferdous Ur Rahaman, Abed Hussain Khan, Md Nazmul Hasan, Shahriar Ahmed, Mohammod Jobayer Chisti, Tahmeed Ahmed

**Affiliations:** 1 Nutrition Research Division, International Centre for Diarrhoeal Disease Research, Bangladesh (icddr,b), Dhaka, Bangladesh; 2 Department of Internal Medicine, Bangabandhu Sheikh Mujib Medical University (BSMMU), Dhaka, Bangladesh; 3 Department of Cardiology, Bangabandhu Sheikh Mujib Medical University (BSMMU), Dhaka, Bangladesh; 4 Infectious Diseases Division, International Centre for Diarrhoeal Disease Research, Bangladesh (icddr,b), Dhaka, Bangladesh

## Abstract

**Background:**

In developing settings, comparative data on COVID hospitalised survivors (HS) and non-hospitalised survivors (NHS) is scarce. We determined burdens, incidence, evolution, and associated factors of long COVID-19 over two years among these groups.

**Methods:**

We conducted a longitudinal cohort study in Dhaka, Bangladesh, and recruited confirmed COVID-19 survivors from December 2020 to May 2021 (previously reported). 346 survivors underwent in-person follow-ups at five, nine, 18 months and two years post-infection. The assessment included long COVID symptoms, cardiorespiratory function, neuropsychiatric conditions, quality of life, and laboratory tests. The outcomes included one or more symptoms and/or signs indicative of long COVID, aligning closely with the World Health Organization definition of post-COVID-19 condition.

**Results:**

Of the 346 participants, we included 326 in the analysis. 78% of HS (n/N = 171/219) and 62% of NHS (n/N = 55/89) reported at least one sequela symptom. HS had higher odds of palpitations, headaches, dizziness, sleeping difficulties, brain fog, muscle weakness, joint pain, hypertension, insulin requirement, poor quality of life, and prolonged corrected QT intervals on electrocardiogram compared to NHS at two years (95% confidence interval (CI)>1). Regarding evolution, sequelae-symptoms, neurological outcomes, restrictive spirometry findings, and electrocardiogram abnormalities remained unchanged, although psychiatric sequelae, quality of life, and exercise capacity improved in both groups. Hospital readmission rates significantly increased (*P* < 0.05). The incidence rates of palpitations, cough, and hypertension were higher in HS compared to NHS (95% CI>1). Two or more vaccine doses decreased the risk of respiratory (adjusted risk ratio (aRR) = 0.76; 95% CI = 0.63–0.91) and psychiatric sequelae (aRR = 0.78; 95% CI = 0.66–0.92) than one or no doses.

**Conclusions:**

COVID-19 survivors, particularly HS, experienced a higher burden of persistent symptoms and health issues two years after infection. However, vaccination significantly reduced the risk of respiratory and psychiatric outcomes. These findings highlight the importance of ongoing vaccination programs and the need for targeted rehabilitation services in low-resource settings.

Post-COVID syndrome, or long COVID, refers to the long-term health consequences of COVID-19 that can persist for months or even years after recovering from the acute infection [1,2]. Despite a significant decline in COVID-19 cases and the disease no longer being a global health emergency, new cases are still being reported. Between 4–31 March 2024, 98 countries reported COVID-19 cases, and 39 countries reported deaths. Thus, long COVID has emerged as a substantial public health issue, necessitating a comprehensive and patient-centred approach.

A recent study demonstrated that in 12.7% of patients, long COVID symptoms can be attributed to COVID-19 [3]. Thus, there would be more than 100 million cases of long COVID among the 775 million documented COVID-19 cases as of May 2023 (World Health Organization (WHO) report). The actual number is likely much higher due to a lack of documentation, particularly in low- and middle-income countries (LMICs). Long COVID is a multisystem condition that can profoundly affect individuals’ health and well-being [4,5]. However, as long as COVID can evolve, it is imperative to gain a better understanding of long-term clinical outcomes to design future intervention studies aimed at preventing or alleviating the suffering of such patients. Most studies have reported the evolution of symptoms from three to 12 months [6–13].

A recent longitudinal study revealed that even after two years, 55% of participants continued to experience at least one symptom, although a notable improvement trend was observed from six months onwards. However, the study focused on hospitalised participants, making it challenging to extrapolate findings to non-hospitalised patients [14]. Some other studies have also provided long-term follow-up data extending up to two years. However, these studies were constrained by their cross-sectional or retrospective design, reliance on phone interviews, lack of comprehensive in-person follow-ups, physical examination, and investigations, and inclusion of only hospitalised patients or a subgroup experiencing lingering symptoms [15–17]. No studies with long-term follow-up have directly compared outcomes between hospitalised and non-hospitalised individuals. Furthermore, most studies have been conducted in developed countries, resulting in a dearth of data from densely populated LMICs where individuals may have been badly affected by long-term COVID-19. Therefore, there is an urgent need for longitudinal studies comparing outcomes between hospitalised survivors (HS) and non-hospitalised survivors (NHS) to enhance our understanding of long COVID, particularly in LMICs.

We sought to determine the burden of symptoms, as well as clinical and laboratory outcomes, in HS and NHS between five months to two years after acute COVID-19, aiming to capture the ongoing evolution of long COVID in LMICs. We reported the incidence of long COVID outcomes and severe acute respiratory syndrome coronavirus 2 (SARS-CoV-2) specific antibodies at two years and analysed the association between sociodemographic characteristics and the impact of hospitalisation on long COVID.

## METHODS

### Study design

We carried out a prospective, longitudinal study in Dhaka, Bangladesh. The participants were COVID-19 survivors (aged >18 years) who visited the study hospitals with or without the hospitalisation requirement. Eligibility criteria and methods have been described previously [18]. In brief, we included in the study COVID-19 HS who demonstrated significant symptom improvement for three consecutive days leading to their discharge, as well as NHS who showed significant symptom improvement for three consecutive days and completed the isolation period of at least 10 days. We excluded participants with pre-existing mental illnesses or those residing outside the Dhaka district. We conducted in-person follow-ups after one month (baseline), three months, and five months of recovery from acute COVID-19, and the findings have been described previously. We screened a total of 1981 confirmed COVID-19 cases listed in the registry of the study hospitals between 15 December 2020 and 30 May 2021. Of these, we invited 622 survivors to participate, and 362 were enrolled after obtaining written informed consent. Of the 362 enrolled cases, 346 completed the five-month follow-up and were subsequently invited to continue follow-ups at nine and 18 months, and two years. Ethical approval was obtained from the institutional review boards of the study sites at the International Centre for Diarrhoeal Disease Research, Bangladesh (icddr,b,) and Bangabandhu Sheikh Mujib Medical University Hospital (BSMMU).

### Follow-up procedures

Participants visited the follow-up clinics at Dhaka Hospital of icddr,b, and BSMMU for face-to-face interviews (Methods S1 in the **Online Supplementary Document**). At each visit, study clinicians conducted detailed interviews and physical examinations, including objective assessments of smell, taste, and muscle strength, as well as laboratory tests. To assess cardiorespiratory function, we used a six-minute walk test, modified Medical Research Council (mMRC) dyspnoea scale, and electrocardiogram (ECG) at all visits. An echocardiogram was performed on HS at the five and 24-month time points. Neuropsychiatric assessment tools included the UK screening test for peripheral neuropathy, the Mini-Mental State Examination for cognitive function, the Primary Care Post-Traumatic Stress Disorder (PTSD) Screen for PTSD, and the WHO Self-Reporting Questionnaire for identifying depression and anxiety-related disorders. Starting at nine months, we used the Patient Health Questionnaire-9 to evaluate depressive symptoms experienced over the previous 14 days [19].

We applied the Chalder Fatigue Scale (CFQ-11), an 11-item self-reported questionnaire, to measure the extent and severity of fatigue over the past month. A score ≥4 indicates severe fatigue (range zero to 11) [20]. The core research team consisted of expert clinicians specialised in infectious diseases, internal medicine, and cardiology. They convened weekly discussions/meetings on a virtual platform to discuss challenges, address individual medical needs, and recommend specific management as necessary. A psychiatrist assessed all participants with psychiatric symptoms. Counselling and treatment for psychiatric, cardiologic, and respiratory conditions were crucial elements of the study.

### Lung function and serological tests

At each follow-up visit, we performed a lung function test using spirometry [18] and we collected blood and urine samples for a complete blood count, serum alanine transaminase, serum creatinine, random blood glucose, and urine routine microscopy test. Additionally, to determine receptor-binding domain (RBD) specific immunoglobulin G (IgG) antibody concentrations at the two-year time point, we performed an enzyme-linked immunosorbent assay test that employed a monoclonal antibody, specifically targeting IgG against the spike RBD of SARS-CoV-2. We established a threshold of 500 ng/mL anti-RBD specific IgG as the cutoff for seropositivity. To establish this cutoff, we utilised the upper limit of the SARS-CoV-2 IgG antibodies from a selection of 200 randomly chosen pre-pandemic serum samples collected from various regions of Bangladesh [21].

### Outcome measures

The outcomes comprised one or more symptoms and/or signs indicative of long COVID, aligning closely with the WHO definition of post-COVID-19 condition, as previously described [22]. These symptoms and/or signs included: 1) self-reported symptoms of COVID-19 (persistent symptoms lasting at least two months or relapsing symptoms), 2) neurological findings, 3) psychiatric sequelae (PTSD, depression, anxiety disorder, chronic fatigue, or cognitive impairment), 4) respiratory findings, 5) cardiovascular findings, 6) muscle weakness, 7) poor quality of life, and 8) new requirement for insulin for glycaemic control. Detailed outcome measures are presented in Methods S1 in the **Online Supplementary Document**.

### Statistical analysis

We presented the sociodemographic characteristics, sequelae symptoms, and findings at five, nine, and 18 months, as well as two-year follow-ups as frequencies and medians (MDs) and interquartile ranges (IQRs). We utilised a doubly robust propensity score matching method to balance the baseline characteristics between the HS and NHS groups. This was achieved using a 1:1 greedy nearest neighbour algorithm with a calliper width 0.2 [18].

To compare the sociodemographic and clinical characteristics between the groups, we applied χ^2^, Fisher exact, or Mann-Whitney U tests as applicable. To compare the relative risk of long COVID or post-COVID syndrome occurrence in HS compared to NHS at all visits, we performed multivariable robust Poisson regression models with robust variance. We built individual models for each outcome, adjusting for all covariates (age, sex, body mass index (BMI), group, site, and comorbidity) in the propensity score matched cohort (Methods S1 in the **Online Supplementary Document**).

To assess the temporal trajectory of long COVID among survivors from five months to two years, we employed similarly adjusted generalised estimating equation (GEE) models. To capture the evolution of outcomes between hospitalised and non-hospitalised groups, we included an interaction term between groups and time. We calculated the incidence rate (occurrence of new sequelae symptoms or physical and laboratory outcomes from five months to two years and the rate per 1000 person-years) among survivors. We applied Poisson regression models with robust variance to compare the incidence rates between the cohorts while adjusting for age, sex, BMI, site, and comorbidity. We used GEE models to identify risk factors for sequelae symptoms, respiratory, cardiovascular, neurological, psychiatric outcomes, and chronic fatigue at the two-year time point. The models included age, sex, group, diabetes, cigarette smoking, site, occupation, and COVID-19 vaccination status to determine predictors of long COVID among survivors. All hypothesis tests were two-sided, with statistical significance set at a *P*-value <0.05. The statistical analyses were carried out using Stata, version 15.0 (StataCorp LLC, College Station, Texas, USA).

## RESULTS

Of the 346 participants who completed five-month visits, 14 (4%) were lost to follow-up, four (1%) died, and 20 (6%) developed reinfection with COVID-19. Thus, we included in the analysis 326 participants. Of 326 participants, 313 (96%), 312 (96%), and 308 (95%) survivors completed follow-ups at nine months, 18 months, and two years, respectively (Figure S1 in the **Online Supplementary Document**). The follow-up period was from April 2021 to June 2023. Adequate propensity score matching was achieved, with standardised mean differences of <10% for all baseline characteristics (Table S1 in the **Online Supplementary Document**).

Of the 326 survivors, the MD age was 50 years (IQR = 39–60). HS were significantly older than the NHS. 62% of 326 participants were male. 68% (n/N = 223/326) reported at least one comorbidity, with the most common being hypertension (50%) and diabetes (37%). MD RBD-specific IgG concentrations at the two-year time point were 9504 (IQR = 5962–31 375). 93% (n/N = 304/326) of survivors received two or more doses of COVID-19 vaccination. The MD duration from COVID-19 diagnosis (positive reverse transcription-polymerase chain reaction) to the first dose of vaccine was 109 days (IQR = 60–196). The MD follow-up period after symptom onset was 171 days (IQR = 165–178) at five months, 293 days (IQR = 289–301) at nine months, 566 days (IQR = 559–572) at 18 months, and 749 days (IQR = 741–757) at two years (**Table 1**).

**Table 1:** Baseline characteristics of hospitalised and non-hospitalised COVID-19 survivors in Dhaka, Bangladesh*

Characteristics	Total (n = 326)	Non-hospitalised (n = 95)	Hospitalised (n = 231)	*P-*value
Age in years, MD (IQR)	50.00 (39.00–60.00)	40.00 (32.00–51.00)	55.00 (43.00–62.00)	<0.001
Male	203 (62)	55 (58)	148 (64)	0.296
Years of schooling				
*12*	71 (22)	19 (20)	52 (23)	0.618
*≤16*	105 (32)	32 (34)	73 (32)	0.715
*>16*	150 (46)	44 (46)	106 (46)	0.944
Occupation				
*Employed*	199 (61)	61 (64)	138 (60)	0.452
*Unemployed*	87 (27)	28 (29)	59 (26)	0.466
*Retired*	40 (12)	6 (6)	34 (15)	0.036
BMI in kg/m^2^, MD (IQR)	27.04 (24.13–29.88)	26.99 (24.06–30.33)	27.21 (24.15–29.78)	0.783
≥2 doses of COVID-19 vaccines	304 (93)	89 (94)	215 (93)	0.842
Duration from COVID-19 diagnosis to first dose of vaccine in days, MD (IQR)	109.00 (60.00–195.50)	114.00 (71.00–237.00)	107.00 (56.00–178.00)	0.052
BSMMU site	121 (37)	23 (24)	98 (42)	0.002
Cigarette smoking	48 (15)	19 (20)	29 (13)	0.085
At least one comorbidity	223 (68)	48 (51)	175 (76)	<0.001
Hypertension	163 (50)	27 (28)	136 (59)	<0.001
Ischemic heart disease	52 (16)	12 (13)	40 (17)	0.294
Chronic liver disease	8 (2)	1 (1)	7 (3)	0.269
Diabetes	122 (37)	19 (20)	103 (45)	<0.001
Hypothyroidism	32 (10)	9 (9)	23 (10)	0.894
Chronic kidney disease	18 (6)	3 (3)	15 (6)	0.231
Immunocompromised conditions	11 (3)	3 (3)	8 (3)	0.595
Stroke	16 (5)	1 (1)	15 (6)	0.028
Duration in days from symptom onset to 5 months, MD (IQR)	171.00 (165.00–178.00)	172.00 (167.00–177.00)	170.00 (164.00–179.00)	0.373
Duration in days from symptom onset to 9 months, MD (IQR)	293.00 (289.00–301.00)	295.00 (291.00–303.00)	292.00 (287.00–300.00)	0.032
Duration in days from symptom onset to 18 months, MD (IQR)	566.00 (559.00–572.00)	568.00 (562.00–573.00)	564.00 (558.00–571.00)	0.012
Duration in days from symptom onset to 2 years, MD (IQR)	749.00 (741.00–757.00)	751.00 (746.00–756.00)	746.00 (740.00–758.00)	0.028
Diagnosis during acute COVID-19				
*Mild*	80 (25)	80 (84)	0 (0)	<0.001
*Moderate*	89 (27)	15 (16)	74 (32)	0.003
*Severe*	71 (22)	0 (0)	71 (31)	<0.001
*Critical*	86 (26)	0 (0)	86 (37)	<0.001
Number of family members affected by COVID-19, MD (IQR)	2 (1–3)	2 (1–3)	2 (1–3)	0.260
Lost a family member due to COVID-19	58 (18)	14 (15)	44 (19)	0.355
Treatment during acute COVID-19				
*Antibiotics*	309 (95)	84 (88)	225 (97)	0.001
*Antivirals*	199 (61)	70 (74)	129 (56)	0.003
RBD-specific IgG concentrations in ng/ml, MD (IQR)	9503.80 (5962.11–31 374.76)	9222.99 (7480.78–25 155.30)	9506.67 (2615.66–33 613.74)	0.455
RBD-specific IgG>500 ng/ml, n/N	308/309 (99.7)	89/89 (100)	219/220 (99.5)	0.712

BMI – body mass index, BSMMU – Bangabandhu Sheikh Mujib Medical University, icddr,b – International Centre for Diarrhoeal Disease Research, Bangladesh, IgG – immunoglobulin G, IQR – interquartile range, MD – median, RBD – receptor binding domain

*Presented as n (%) unless specified otherwise.

78% of HS (n/N = 171/219) and 62% of NHS (n/N = 55/89) reported at least one sequela symptom two years after acute COVID-19. Although the proportion of participants with any sequelae symptom decreased from 81% (n/N = 264/326) at five months to 73% at two years, it largely remained consistent for both groups, and the prevalence remained comparable between the groups at all time points. The multivariable robust Poisson regression models revealed that even at two year-visit, HS more often reported palpitation (adjusted risk ratio (aRR) = 2.83; 95% confidence interval (CI) = 1.03–7.81), headache (aRR = 1.64; 95% CI = 1.13–2.37), dizziness (aRR = 2.52; 95% CI = 1.47–4.31), sleeping difficulty (aRR = 1.42; 95% CI = 1.05–1.93), brain fog (aRR = 1.56; 95% CI = 1.04–2.25), muscle weakness (aRR = 1.60; 95% CI = 1.14–2.25), and joint pain (aRR = 1.33; 95% CI = 1.02–1.73) than the NHS. Regarding the evolution, most sequelae symptoms remained unchanged. However, in the NHS group, the proportion of participants with palpitations, dizziness, and loss of appetite significantly decreased, and in the HS group, headache, and loss of appetite significantly decreased from five months to two years (**Table 2**; Table S2 in the **Online Supplementary Document**).

**Table 2:** Prevalence and evolution of clinical outcomes among non-hospitalised and hospitalised COVID-19 survivors over time*

	5-month visit			9-month visit			18-month visit			2-year visit			*P*-value†	*P*-value†
**Outcome**	**NHS (n = 95)**	**HS (n = 231)**	**aRR (95% CI)**	**NHS (n = 91)**	**HS (n = 222)**	**aRR (95% CI)**	**NHS (n = 91)**	**HS (n = 221)**	**aRR (95% CI)**	**NHS (n = 89)**	**HS (n = 219)**	**aRR (95% CI)**	**NHS (n = 95)**	**HS (n = 231)**
Any symptom sequelae	71 (75)	193 (84)	1.07 (0.93–1.23)	76 (84)	200 (90)	1.05 (0.95–1.16)	72 (79)	185 (84)	1.06 (0.94–1.20)	55 (62)	171 (78)	1.16 (0.99–1.36)	0.222	0.330
*Fatigue*	42 (44)	122 (53)	1.11 (0.84–1.45)	40 (44)	132 (59)	1.30 (1.08–1.57)	44 (48)	116 (52)	1.07 (0.83–1.38)	26 (29)	92 (42)	1.25 (0.85–1.83)	0.219	0.067
*Palpitation*	16 (17)	47 (20)	1.02 (0.60–1.71)	14 (15)	34 (15)	1.01 (0.54–1.88)	10 (11)	34 (15)	1.14 (0.57–2.30)	4 (4)	31 (14)	2.83 (1.03–7.81)	0.020	0.129
*Chest pain*	7 (7)	31 (13)	1.56 (1.01–2.43)	15 (16)	39 (18)	1.04 (0.60–1.81)	8 (9)	28 (13)	1.15 (0.53–2.50)	7 (8)	20 (9)	1.0 (0.4–2.4)	0.730	0.073
*Breathing difficulty*	7 (7)	24 (10)	1.64 (1.04–2.57)	7 (8)	24 (11)	1.38 (0.63–3.06)	7 (8)	36 (16)	1.55 (0.68–3.55)	6 (7)	35 (16)	1.47 (0.62–3.51)	0.955	0.035
*Cough*	7 (7)	34 (15)	1.63 (1.07–2.48)	20 (22)	70 (32)	1.43 (1.09–1.87)	20 (22)	57 (26)	1.12 (0.70–1.79)	9 (10)	36 (16)	1.30 (0.64–2.67)	0.653	0.897
*Headache*	23 (24)	71 (31)	1.36 (1.05–1.75)	34 (37)	71 (32)	0.91 (0.64–1.30)	29 (32)	59 (27)	0.97 (0.64–1.47)	11 (12)	45 (21)	1.64 (1.13–2.37)	0.144	0.021
*Dizziness*	13 (14)	50 (22)	1.23 (0.67–2.29)	19 (21)	43 (19)	0.94 (0.58–1.53)	10 (11)	44 (20)	1.52 (1.06–2.19)	4 (4)	33 (15)	2.52 (1.47–4.31)	0.040	0.119
*Sleeping difficulty*	23 (24)	83 (36)	1.17 (0.78–1.76)	22 (24)	75 (34)	1.12 (0.74–1.71)	24 (26)	74 (33)	1.13 (0.76–1.68)	14 (16)	61 (28)	1.42 (1.05–1.93)	0.297	0.128
*Brain fog*	13 (14)	41 (18)	1.10 (0.60–2.02)	11 (12)	41 (18)	1.36 (0.72–2.55)	11 (12)	29 (13)	1.02 (0.50–2.10)	7 (8)	29 (13)	1.56 (1.04–2.25)	0.312	0.115
*Loss of appetite*	15 (16)	46 (20)	1.10 (0.64–1.91)	9 (10)	47 (21)	1.86 (1.28–2.71)	9 (10)	41 (19)	1.46 (0.70–3.01)	4 (4)	23 (11)	1.85 (0.68–5.09)	0.010	0.012
*Body ache*	19 (20)	56 (24)	1.12 (0.68–1.84)	24 (26)	61 (27)	1.01 (0.66–1.53)	26 (29)	63 (29)	0.99 (0.67–1.47)	15 (17)	43 (20)	1.05 (0.62–1.80)	0.803	0.263
*Muscle weakness*	13 (14)	45 (19)	1.18 (0.64–2.18)	13 (14)	53 (24)	1.62 (1.16–2.26)	22 (24)	64 (29)	1.07 (0.69–1.67)	11 (12)	58 (26)	1.60 (1.14–2.25)	0.728	0.101
*Joint pain*	21 (22)	85 (37)	1.54 (1.19–1.98)	34 (37)	94 (42)	1.09 (0.79–1.51)	21 (23)	93 (42)	1.61 (1.06–2.46)	20 (22)	89 (41)	1.33 (1.02–1.73)	0.581	0.651
Neurologic sequelae, n/N	19/94 (20)	93/224 (42)	1.64 (1.07–2.51)	19/86 (22)	80/211 (38)	1.39 (0.94–2.06)	27/87 (31)	85/210 (40)	1.18 (0.83–1.66)	26/83 (31)	93/206 (45)	1.30 (0.92–1.85)	0.132	0.682
*Peripheral neuropathy*	17 (18)	79 (35)	1.48 (1.13–1.94)	18 (21)	66 (31)	1.18 (0.77–1.82)	23 (26)	65 (31)	1.07 (0.72–1.58)	24 (29)	72 (35)	1.12 (0.75–1.68)	0.159	0.935
*Anosmia*	2 (2)	6 (3)	1.06 (0.26–4.34)	0 (0)	5 (2)	NA	1 (1)	3 (1)	0.90 (0.10–8.11)	0 (0)	6 (3)	NA	0.237	0.620
*Absent/impaired taste*	1 (1)	4 (2)	1.00 (0.96–1.03)	0 (0)	5 (2)	NA	1 (1)	6 (3)	1.00 (0.91–1.09)	0 (0)	3 (1)	NA	0.953	0.965
*Tremor*	1 (1)	9 (4)	1.83 (0.37–9.11)	0 (0)	11 (5)	NA	3 (3)	18 (9)	2.09 (0.69–6.38)	5 (6)	21 (10)	1.05 (0.45–2.46)	0.031	0.003
Psychiatric sequelae	42 (44)	108 (47)	1.06 (0.81–1.39)	39 (43)	102 (46)	1.08 (0.80–1.45)	33 (36)	81 (37)	1.04 (0.74–1.46)	20 (22)	65 (30)	1.16 (0.71–1.91)	0.003	<0.001
*Depression/anxiety disorder*	38 (40)	101 (44)	1.09 (0.82–1.46)	33 (36)	94 (42)	1.11 (0.81–1.53)	31 (34)	74 (33)	0.96 (0.68–1.36)	17 (19)	54 (25)	1.11 (0.67–1.84)	0.014	<0.001
*Posttraumatic stress disorder*	14 (15)	16 (7)	0.47 (0.24–0.93)	4 (4)	11 (5)	1.04 (0.39–2.76)	0 (0)	4 (2)	NA	NA	NA	NA	NA	NA
*Cognitive impairment*	1 (1)	15 (6)	2.07 (0.27–15.86)	7 (8)	14 (6)	0.87 (0.27–2.76)	3 (3)	12 (5)	1.07 (0.34–3.40)	4 (4)	17 (8)	1.43 (0.52–3.94)	0.172	0.153
*Chronic fatigue (chalder bimodal score ≥4)*	NA	NA	NA	45 (49)	119 (54)	1.05 (0.80–1.38)	45 (49)	123 (56)	1.11 (0.82–1.50)	29 (33)	98 (45)	1.09 (0.76–1.58)	0.357	0.004
*Physical fatigue (score Q1–Q7)*	NA	NA	NA	5.55 (1.79)	5.92 (1.99)	NA	5.33 (1.65)	5.54 (1.74)	NA	5.16 (1.26)	5.53 (1.37)	NA	0.056	0.001
*Psychological fatigue (score Q8–Q11)*	NA	NA	NA	9.98 (3.17)	10.90 (3.75)	NA	9.89 (3.07)	10.75 (3.68)	NA	9.00 (2.51)	9.61 (2.95)	NA	0.009	<0.001
Respiratory sequelae, n/N	29 (31)	104 (45)	1.30 (0.92–1.82)	29/87 (33)	87/219 (40)	1.12 (0.81–1.54)	23/88 (26)	78/218 (36)	1.28 (1.00–1.62)	21/85 (25)	61/212 (29)	1.10 (0.71–1.71)	0.265	0.001
*mMRC grade ≥2*	26 (27)	83 (36)	1.21 (0.83–1.77)	24 (28)	80 (36)	1.21 (0.84–1.73)	19 (22)	71 (33)	1.39 (1.07–1.81)	18 (21)	53 (25)	1.06 (0.64–1.75)	0.216	0.014
*Bronchial/diminished breath sound*	2 (2)	16 (7)	1.07 (0.27–4.31)	0 (0)	3 (1)	NA	0 (0)	1 (0.5)	NA	0 (0)	0 (0)	NA	0.973	<0.001
*Tachypnoea*	5 (5)	20 (9)	1.17 (0.46–2.95)	12 (14)	16 (7)	0.51 (0.25–1.02)	9 (10)	20 (9)	0.98 (0.47–2.08)	6 (7)	13 (6)	0.87 (0.34–2.23)	0.908	0.409
Cardiovascular sequelae, n/N	29 (31)	90 (39)	1.10 (0.77–1.57)	23/87 (26)	99/219 (45)	1.29 (1.01–1.63)	15/88 (17)	69/218 (32)	1.54 (1.15–2.05)	16/85 (19)	80/212 (38)	1.38 (1.04–1.83)	0.044	0.255
*Hypertension*	19 (20)	50 (22)	1.06 (0.66–1.70)	16 (18)	71 (32)	1.64 (1.24–2.16)	11 (13)	50 (23)	1.77 (1.27–2.47)	11 (13)	56 (26)	1.57 (1.12–2.19)	0.145	0.884
*Tachycardia*	10 (11)	29 (13)	1.16 (0.59– 2.30)	7 (8)	23 (11)	1.30 (0.59– 2.85)	2 (2)	8 (4)	1.61 (0.35– 7.45)	2 (2)	6 (3)	1.0 (0.2– 5.4)	0.021	<0.001
*Oedema*	4 (4)	30 (13)	1.76 (0.76– 4.21)	4 (5)	26 (12)	2.18 (1.28–3.71)	2 (2)	29 (13)	3.45 (1.66–7.19)	6 (7)	33 (16)	1.63 (1.03–2.57)	0.580	0.592
Muscle weakness‡	1 (1)	25 (10)	5.31 (1.92– 14.70)	9 (10)	21 (9)	1.17 (0.45–3.03)	8 (9)	21 (10)	1.05 (0.48–2.30)	4 (4)	25 (11)	1.99 (0.75–5.33)	0.649	0.263
Required insulin, n/N§	2/19 (11)	58/104 (56)	4.77 (1.26– 18.01)	6/20 (30)	73/101 (72)	2.42 (1.26– 4.64)	5/20 (25)	76/101 (75)	2.76 (1.30–5.85)	7/20 (35)	74/100 (74)	1.96 (1.09–3.49)	0.056	0.118
Poor quality of life	36 (38)	101 (44)	1.13 (0.82– 1.57)	29 (32)	80 (36)	1.09 (0.75–1.58)	26 (29)	73 (33)	1.04 (0.70–1.55)	16 (18)	73 (33)	1.47 (1.10–1.96)	0.001	0.004
*Distance in meters walked in 6 minutes, MD (IQR)*	358 (315–388)	352 (300–403)	NA	366 (329–410)	373 (320–420)	NA	432 (388–470)	410 (345–465)	NA	444 (412–480)	420 (364–465)	NA	<0.001	<0.001
Health care use														
*Received outpatient care in last 7 days of follow-up visit*	24 (25)	70 (30)	1.19 (0.77–1.84)	30 (33)	66 (30)	0.99 (0.66–1.48)	32 (35)	72 (33)	0.85 (0.58–1.24)	36 (40)	83 (38)	0.99 (0.73–1.34)	0.079	0.181
*Required hospitalisation*	1 (1)	5 (2)	1.46 (0.15–14.41)	4 (4)	9 (4)	1.19 (0.39–3.58)	2 (2)	14 (6)	2.25 (0.67–7.49)	3 (3)	7 (3)	1.07 (0.53–2.13)	0.007	<0.001

aRR – adjusted risk ratio, CI – confidence interval, HS – hospitalised survivors, IQR – interquartile range, MD – median, NHS – non-hospitalised survivors, NA– not applicable, Q – question, mMRC – modified Medical Research Council

*Presented as n (%) unless specified otherwise. All estimates are adjusted for age, sex, body mass index, any comorbidity and group (non-hospitalized/hospitalised), and site (icddr,b/BSMMU).

†*P*-value is for the interaction between the follow-up visit and the groups. *P*-value is derived from the generalised estimating equation (GEE) models, indicating the trend of changing characteristics over time. The pooled risk ratios from GEE models are not shown.

‡The Medical Research Council (MRC) scale for muscle strength was applied to determine muscle weakness.

§Among subsamples with diagnosed diabetes. The complete list of reported symptoms is shown in Table S2 in the **Online Supplementary Document**.

The estimated risk of any neurological findings (aRR = 1.64; 95% CI = 1.07–2.51), particularly peripheral neuropathy (aRR = 1.48; 95% CI = 1.13–1.94), was higher among HS at five-month visits compared to non-hospitalised groups. Although the evolution of any neurological findings, including peripheral neuropathy, remained unremarkable, the tremors significantly increased from five months to two years (3–8%) among HS (*P* = 0.003) and NHS (*P* = 0.031). The prevalence of psychiatric sequelae decreased significantly from 46% at five months to 45% at nine months, 37% at 18 months, and 28% at two-year visits in both HS (*P* < 0.001) and NHS (*P* = 0.003). The prevalence of chronic fatigue, as defined by a CFQ-11 bimodal score ≥4, was 47% (n/N = 146/313) at nine months, then gradually declined to 46% (n/N = 144/312) at 18 months and 35% (n/N = 109/308) at two years. However, the evolution of chronic fatigue was significant among HS (*P* = 0.004) but remained stable among NHS (*P* = 0.357). The risk of developing psychiatric sequelae was comparable between the groups, except that the risk of PTSD was significantly less in HS than in NHS at the five-month visit (aRR = 0.47; 95% CI = 0.24–0.93). Regarding depressive symptoms as measured by pHQ9, HS more often had depressive symptoms than NHS at two years (aRR = 1.74; 95% CI = 1.08–2.81) (Table S3 in the **Online Supplementary Document**).

In HS, the prevalence of dyspnoea (mMRC≥2) gradually declined from 36% (n/N = 83/231) at five months to 25% (n/N = 53/212) at two years (*P* = 0.014), while in NHS, the proportion remained unchanged over time (27% (n/N = 26/95) at five months, and 21% (n/N = 18/85) at two years; *P* = 0.216). Although we observed a trend of a higher risk of dyspnoea in the HS, the risk became statistically significant at 18 months (aRR = 1.39; 95% CI = 1.07–1.81) compared to NHS. The risk of experiencing cardiovascular sequelae, particularly hypertension, was more often observed in HS at the nine-month (aRR = 1.64; 95% CI = 1.24–2.16), 18-month (aRR = 1.77; 95% CI = 1.27–2.47), and two years (aRR = 1.57; 95% CI = 1.12–2.19) compared to the NHS. Regarding time trend, the proportion of tachycardia significantly declined in both groups, although the proportion of hypertension and oedema remained stable over time.

Among pre-existing diabetic patients, HS were at higher risk of worsening glycaemic control and required insulin therapy compared to NHS at five-month (aRR = 4.77; 95% CI = 1.26–18.01), nine-month (aRR = 2.42; 95% CI = 1.26–4.64), 18-month (aRR = 2.76; 95% CI = 1.30–5.85), and two-year time points (aRR = 1.96; 95% CI = 1.09–3.49). Although the quality of life improved significantly in both groups from five months to two years, HS was 1.5 times more likely to experience poor quality of life than NHS (aRR = 1.47; 95% CI = 1.10–1.96) at two years. COVID-19 survivors significantly increased the MD six-minute walk distance over time (*P* < 0.001), irrespective of groups. 39% (n/N = 119/308) of participants received outpatient care within seven days of the two-year visit. The proportion of participants requiring hospitalisation during the study period increased significantly over time (NHS *P* = 0.007 and HS *P* < 0.001). The reasons for hospitalisation have been listed in Table S4 in the **Online Supplementary Document**.

**Figure 1:**
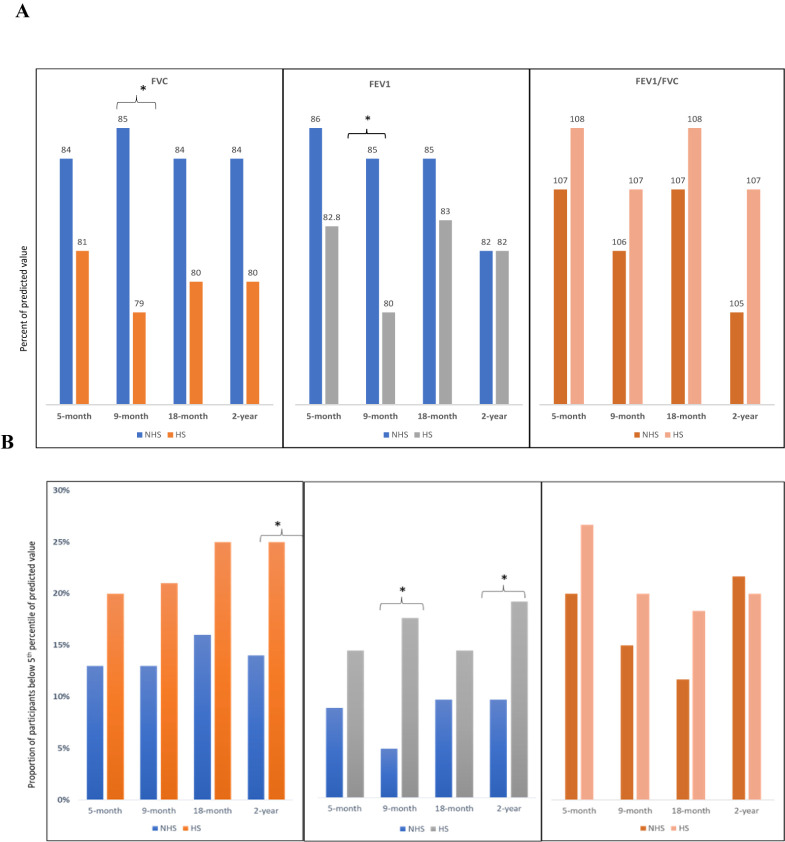
Spirometry findings of hospitalised and non-hospitalised COVID-19 survivors over time. **Panel A.** Percentage of predicted values for spirometry parameters in COVID-19 survivors. **Panel B.** The proportions of participants with spirometry parameters below the fifth percentile of the predicted values. *P < 0.05. FEV1 – forced expiratory volume in one second, FVC – forced vital capacity, FEV1/FVC – forced expiratory volume in one second/forced vital capacity ratio.

At two years, 41% (n/N = 86/212) of HS and 34% (n/N = 29/85) of NHS were anaemic. The evolution of anaemia remained unremarkable throughout the study period among the entire cohort. Normal spirometry findings were 20% less often found among HS compared to NHS at the nine-month (aRR = 0.82; 95% CI = 0.69–0.95) and 18-month (aRR = 0.79; 95% CI = 0.67–0.92) visits. The evolution of spirometry findings, including restrictive patterns, remained unchanged in both cohorts (**Figure 1****,** Panel A–B, **Table 3**). The proportion of participants with at least one ECG abnormality did not evolve significantly over time, with 21% (n/N = 18/85) of NHS and 36% (n/N = 75/209) of HS showing one or more ECG abnormalities at two years. However, there was an increasing trend of bradycardia observed among HS participants from five months (n/N = 7/226, 3%) to two years (n/N = 19/209, 9%). Notably, the ECG findings of HS were more likely to reveal prolonged corrected QT intervals (QTc) than NHS at nine months (aRR = 1.93; 95% CI = 1.23–3.04), 18 months (aRR = 1.83; 95% CI = 1.11–3.02), and two years (aRR = 1.83; 95% CI = 1.10–3.02). Of the 127 HS who underwent an echocardiogram at five months, 109 underwent the test again at two years. The proportion of participants with at least one abnormal echocardiogram finding, particularly pulmonary hypertension and diastolic relaxation abnormality, declined significantly over time (Figure S2 in the **Online Supplementary Document**). The MD pulmonary arterial systolic pressure at two years was 22 mm Hg (IQR = 15–28), significantly lower than the pressure of 32 mm Hg (IQR = 30–37) at five months.

**Table 3:** Laboratory outcomes in non-hospitalised and hospitalised survivors from five months to two years after COVID-19*

	5-month visit		9-month visit		18-month visit		2-year visit		*P*-value†	*P*-value†
Outcomes*	**NHS (n = 95)**	**HS (n = 231)**	**NHS (n = 91)**	**HS (n = 222)**	**NHS (n = 91)**	**HS (n = 221)**	**NHS (n = 89)**	**HS (n = 219)**	**NHS (n = 95)**	**HS (n = 231)**
Anaemia, n/N‡	39/88 (44)	99/224 (44)	35/87 (40)	95/218 (44)	32/88 (36)	85/218 (39)	29/85 (34)	86/212 (41)	0.224	0.283
White blood cell (10^9^/L), MD (IQR)	7.81 (6.63–8.90)	7.74 (6.52–9.18)	7.76 (6.39–9.41)	8.15 (6.81–9.68)	7.42 (6.46–8.71)	7.90 (6.79–9.36)	7.61 (6.45–8.79)	7.64 (6.58–9.33)	0.104	0.644
NL ratio, MD (IQR)	1.86 (1.39–2.28)	1.84 (1.39–2.38)	1.83 (1.44–2.48)	1.98 (1.52–2.53)	1.70 (1.39–2.45)	1.96 (1.49–2.51)	1.93 (1.54–2.54)	1.99 (1.54–2.69)	0.189	0.006
Platelet (10^9^/L), MD (IQR)	263.00 (214.50–306.00)	239.00 (199.00–289.50)	264.00 (223.00–306.00)	246.00 (195.00–306.00)	256.50 (202.50–302.50)	245.50 (200.00–303.00)	264.00 (214.00–300.00)	242.00 (185.00–308.50)	0.307	0.885
High creatinine in mmol/L, n/N§	4 (4)	27 (12)	4/87 (5)	24/217 (11)	8/88 (9)	30/218 (14)	6/85 (7)	24/212 (12)	0.375	0.771
High alanine aminotransferase in IU/L, n/N	20 (21)	52 (23)	15/87 (17)	48/218 (22)	20/88 (23)	41/217 (19)	21/85 (25)	36/212 (17)	0.327	0.205
Random blood glucose in mmol/L, MD (IQR)	6.00 (5.40–7.30)	7.30 (5.90–9.30)	6.50 (5.70–8.10)	7.50 (5.90–10.00)	6.45 (5.70–8.35)	7.45 (6.00–10.10)	5.80 (5.00–8.10)	7.10 (5.70–9.40)	0.454	0.810
Spirometry findings¶										
*Normal, n/N*	69/92 (75)	149/223 (67)	65/87 (75)	137/217 (63)	62/83 (75)	132/214 (62)	59/84 (70)	125/207 (60)	0.937	0.579
*Restrictive*	12 (13)	39 (17)	11 (13)	42 (19)	13 (16)	48 (22)	11 (13)	46 (22)	0.898	0.598
*Obstructive*	11 (12)	35 (16)	11 (13)	38 (18)	8 (10)	34 (16)	14 (17)	36 (17)	0.629	0.802
*Proteinuria, n/N*║	6 (6)	30 (13)	6/87 (7)	19/216 (9)	2/86 (2)	24/217 (11)	4/85 (5)	23/212 (11)	0.367	0.528
ECG findings										
*Any abnormality in ECG, n/N*	26/93 (28)	85/226 (38)	17/87 (20)	72/217 (33)	22/85 (26)	71/217 (33)	18/85 (21)	75/209 (36)	0.436	0.526
*Sinus tachycardia*	1 (1)	11 (5)	2 (2)	7 (3)	3 (4)	5 (2)	0 (0)	7 (3)	0.940	0.320
*Sinus bradycardia*	5 (5)	7 (3)	6 (7)	14 (6)	8 (9)	12 (6)	5 (6)	19 (9)	0.830	0.030
*Ischemic changes*	14 (15)	43 (19)	3 (3)	29 (13)	6 (7)	30 (14)	7 (8)	25 (12)	0.194	0.038
*PR interval prolonged*	2 (2)	10 (4)	1 (1)	7 (3)	3 (4)	10 (5)	1 (1)	9 (4)	0.867	0.901
*PR interval short*	0 (0)	2 (1)	1 (1)	3 (1)	1 (1)	0 (0)	1 (1)	1 (0.5)	0.413	0.436
Prolonged QTc**	7 (8)	28 (12)	6 (7)	33 (15)	5 (6)	28 (13)	5 (6)	26 (12)	0.489	0.786
*Any echocardiogram findings, n/N*††	NA	108/127 (85)	NA	NA	NA	NA	NA	46/109 (42)	NA	<0.001

ECG – electrocardiogram, HS – hospitalised survivors, NA – not applicable, NHS – non-hospitalised survivors, NL ratio – neutrophil to lymphocyte ratio, PR interval – PR interval of ECG, QTc – corrected QT interval of ECG

*Presented as n (%) unless specified otherwise. Adjusted risk ratio (aRR) and (95% CI)>1. Adjusted for age, sex, body mass index, any comorbidity, site, and group (outpatient or non-hospitalised/inpatient or hospitalised). Only significant estimates are shown in the footnote.

†Interaction between follow-up visit and group, indicating the trend of changing characteristics over time. *P*-value from the generalised estimating equation models adjusted for age, sex, body mass index, any comorbidity, site, and group (non-hospitalised/hospitalised).

‡Haemoglobin (g/dl)<13 for male, and <12 for female.

§High creatinine >106 mmol/L.

¶9-month aRR = 0.82 (95% CI = 0.69–0.95) and 18-month aRR = 0.79 (95% CI = 0.67–0.92).

║18-month aRR = 2.83 (95% CI = 1.35–5.93). One plus or more protein in the urine.

**9-month aRR = 1.93 (95% CI = 1.23–3.04), 18-month aRR = 1.83 (95% CI = 1.11–3.02), and two-year aRR = 1.83 (95% CI = 1.10–3.02).

††An echocardiogram was done only in the hospitalised cohort.

The HS had a higher risk of developing new palpitation (aRR = 1.67; 95% CI = 1.01–2.80), and cough (aRR = 1.43; 95% CI = 1.03–1.99) than the NHS. The incidence rate of any cardiovascular dysfunction (aRR = 1.44; 95% CI = 1.03–2.02), including hypertension, was significantly higher in HS than in NHS. Among the entire cohort, the incidence of new-onset diabetes mellitus was 6.32 per 1000 person-years and was comparable between the groups (*P* = 0.840) (Table S5 in the **Online Supplementary Document**). After adjustment, the multivariable GEE models revealed that older age (>60 years) was independently associated with respiratory, cardiovascular, and neurologic outcomes compared to participants aged <40 years. Being female was an independent risk factor for respiratory (aRR = 1.56; 95% CI = 1.12–2.17), neurological (aRR = 1.73; 95% CI = 1.25–2.38), psychiatric outcomes (aRR = 2.25; 95% CI = 1.60–3.17), and chronic fatigue (aRR = 1.78; 95% CI = 1.23–2.58), compared to being male. The risk of developing respiratory and psychiatric outcomes was 24% (aRR = 0.76; 95% CI = 0.63–0.91) and 22% (aRR = 0.78; 95% CI = 0.66–0.92) lower among individuals who received two or more doses compared to those who were unvaccinated or received one dose of COVID-19 vaccine. Diabetes was associated with cardiovascular (aRR = 1.34; 95% CI = 1.02–1.74), and neurological outcomes (aRR = 1.31; 95% CI = 1.01–1.71), and cigarette smoking for psychiatric outcomes (aRR = 1.56; 95% CI = 1.02–2.37) (Table S6 in the **Online Supplementary Document**).

## DISCUSSION

To our knowledge, this is the longest comprehensive in-person follow-up study among hospitalised and non-hospitalised COVID-19 survivors in developing countries. We examined the trajectories of symptoms, clinical, and laboratory outcomes two years after an acute COVID-19 among survivors of the early waves of the pandemic (December 2020 to May 2021, representing the ancestral and alpha-beta waves) [23] who were not vaccinated during diagnosis.

We found a large proportion of survivors experienced a range of sequelae symptoms, and more than two-thirds of participants reported at least one sequela symptom after two years of COVID-19. Although the evolution of long COVID symptoms varied with relapsing symptoms, at two years, HS more often reported palpitation, headache, dizziness, sleeping difficulty, brain fog, muscle weakness, and joint pain compared to NHS.

We observed an insignificant declining trend of at least one symptom from 81% at five months to 73% at two years; the burden is higher compared to the previously reported prevalence of 55% after two years of COVID-19 [14]. However, most studies with two-year follow-ups were conducted in high-income settings where in-person and telerehabilitation programs played vital roles in combating long COVID [24,25]. These findings highlight the need for comprehensive rehabilitation programs in low-resource settings as recommended by international guidelines [26,27]. However, the psychiatric sequelae, quality of life, and exercise capacity improved over two years, as shown in previous studies [14,28].

Earlier studies indicated a significantly higher risk of cardiovascular events in patients with COVID-19 compared with patients without COVID-19. A retrospective study of a 23 million population revealed that the risk of all cardiovascular outcomes was 66% higher among COVID-19 patients one month after acute disease compared to non-COVID-19 controls [29]. Another retrospective study reported similar findings 12 months after COVID-19. The study also highlighted that cardiovascular outcomes were more pronounced among hospitalised than non-hospitalised survivors [30]. We found that from nine months onwards to two years, the prevalence of hypertension and oedema is several times higher among HS compared to NHS. Even the risk of a new occurrence of palpitation and cardiovascular findings are 67% and 44% higher among HS compared to NHS. Among ECG findings, the increasing trend of bradycardia and two times higher risk of having prolonged QTc in HS compared to NHS is a concern. Given the high reported burden of cardiovascular complications among COVID-19 survivors, these findings highlight the need for regular monitoring and management of cardiovascular risk factors such as hypertension and arrhythmia to alleviate hazards of worse clinical outcomes. Previous follow-up studies reported an increased restrictive impairment in late convalescence. Although we did not perform diffusing capacity of the lungs for carbon monoxide, we found that restrictive spirometry findings did not improve even at two years, indicating that fibrotic abnormalities after recovery from COVID-19 may persist for years [14].

We observed a considerable burden of chronic fatigue, as measured by the CFQ 11 scale, persisting even after two years following acute COVID-19. This finding aligns with systematic reviews examining chronic fatigue within the first year post-COVID, which consistently underscore chronic fatigue as a prominent feature of long COVID. The trajectory of chronic fatigue among NHS corresponds with prior literature, which does not substantiate the influence of critical illness on fatigue outcomes [31,32]. We previously reported that 103 HS and 42 NHS received psychiatric consultation and management based on the findings of the one-month visit. Consequently, we found significantly fewer HS had PTSD at five months, underscoring the importance of targeted rehabilitation programs [18].

Previously we reported that the incidence rate of new DM was higher among HS from one to five months post-COVID-19 period compared to NHS. Over the course of two years, the incidence rate of DM became comparable between the groups. However, the poor glycaemic control and requirement of insulin therapy remained consistently higher among HS than NHS. Our findings align with earlier studies indicating that COVID-19 is associated with a 66% increased risk of new diabetes mellitus and that patients with severe COVID-19 face a greater risk of metabolic disturbances [33,34]. Thus, glucose dysregulation after COVID-19 may persist for two years, and vigilant monitoring of glucose dysregulation is warranted.

Among the risk factors for long COVID, older age and being female are well known [14,18]. However, the impact of vaccination merits distinct insinuation; as such, the vaccination reduces the risk of developing psychiatric and respiratory sequelae among participants. A meta-analysis revealed that the impact of vaccination on long-term COVID symptoms was variable among those who received vaccines after SARS-CoV-2 infection. Approximately 20% reported symptomatic improvement, 20% worsening symptoms, and 50% experienced no vaccine impact on long-term COVID-19 symptoms. However, the included studies followed participants from two weeks to six months. Thus, our findings contribute to the body of evidence regarding the trajectory of long COVID following vaccination. It has been hypothesised that immune dysregulation and the persistence of viral antigens may be responsible for long COVID manifestations. Thus, vaccination may reset the dysregulated immune system. Additionally, the antibody response after vaccination may destroy residual viral reservoirs in the host [35,36].

The strengths of the study include a comprehensive in-person follow-up of hospitalised and non-hospitalized COVID-19 survivors at five, nine, and 18 months and two years after acute illness. The inclusion of a range of symptom criteria [7] and physical examination findings, along with simple assessment tools and laboratory tests could support the development of a care bundle for long COVID follow-up clinics, particularly in low-resource settings.

The limitations of the study included the absence of a control group, meaning the observed symptoms and findings may not be specific to COVID-19. Also, we did not include participants infected during the delta, and omicron-driven waves, thus limiting our results to the consequences of the earlier waves consistent with the ancestral and alpha-beta variants. Recent data suggest that the long-term sequelae of these variants are less severe and shorter than those of the ancestral variant [37,38]. Third, like most long COVID studies, we used several self-reported symptom sequelae and scales to measure health outcomes, which might introduce recall bias. Finally, the study was conducted in urban settings of a developing country, which might limit its generalisability to rural or developed settings.

## CONCLUSIONS

COVID-19 survivors experienced a high burden of symptoms at two years, and HS more often had relapsing symptoms than NHS. The burden of hypertension, oedema, poor glycaemic control requiring insulin, and prolonged QTc intervals was higher among HS than NHS throughout the two years. Psychiatric sequelae, quality of life, and exercise capacity improved in both groups, while respiratory sequelae declined in HS and cardiovascular sequelae in NHS. Our findings underscore the need for rehabilitation facilities in low-resource settings to mitigate the consequences of long COVID, particularly in elderly individuals and women. The evidence suggests that vaccination, even in infected individuals, could reduce the burden of long COVID. Our findings emphasize the need for continued vaccination programs and targeted rehabilitation services in low-resource settings.

## Additional material


Online Supplementary Document
