# Supplementary material for: Evolution of long COVID over two years in hospitalised and non-hospitalised survivors in Bangladesh: a longitudinal cohort study
Source: J Glob Health. 2025 Mar 14;15:04075. doi: 10.7189/jogh.15.04075 (PMC11907736; doi:10.7189/jogh.15.04075)
Supplement: Online Supplementary Document [file jogh-15-04075-s001.pdf]

Supplement to: Afroze F, Arafat SM, Ahmed CM, Alam B, Banu S, Islam MZ, Mahfuz M, Quadri F, BhuiyanTR, Parvin I, Ackhter MM, Islam F, Sultana M, Sultana E, Rahaman MFU, Khan AH, Hasan MN, Ahmed S,Chisti MJ, Ahmed T. Evolution of long COVID over two years in hospitalised and non-hospitalisedsurvivors in Bangladesh: a longitudinal cohort study. J Glob Health. 2025;15:04075.

ONLINE SUPPLEMENTARY DOCUMENT

Title: Evolution of long COVID over two years in hospitalized and non-hospitalized survivors in Bangladesh: A longitudinal cohort study

Farzana Afroze[1], Shohael Mahmud Arafat[2], Chowdhury Meshkat Ahmed[3], Baharul Alam[1], Sayera Banu[4], Md. Zahidul Islam[1], Mustafa Mahfuz[1], Firdausi Qadri[4], Taufiqur Rahman Bhuiyan[4], Irin Parvin[1], Mst. Mahmuda Ackhter[1], Farhana Islam[1], Monjeline Sultana[1], Eva Sultana[1], Mohammad Ferdous Ur Rahaman[2], Abed Hussain Khan[2], Md. Nazmul Hasan[2], Shahriar Ahmed[4], Mohammod Jobayer Chisti[1*], Tahmeed Ahmed[1]

[1]Nutrition and Clinical Services Division, International Centre for Diarrhoeal Disease Research, Bangladesh (icddr,b), Dhaka, Bangladesh

[2]Department of Internal Medicine, Bangabandhu Sheikh Mujib Medical University (BSMMU)

[3]Department of Cardiology, Bangabandhu Sheikh Mujib Medical University (BSMMU)

[4]Emerging Infections, Infectious Diseases Division, International Centre for Diarrhoeal Disease Research, Bangladesh (icddr,b), Dhaka, Bangladesh.

S1. Methods

S1.1 Follow-up visits

1. A symptom checklist was used to record newly developed or persistent symptoms. Participants were asked to describe symptoms only if they were new or worse compared to the period prior to COVID-19.
2. Neurological assessment
   Patients underwent a full neurological assessment including cranial nerves, motor and sensory function, pupillary response, and reflexes. Objective olfactory testing was performed by asking the participants to choose the right odor among 2 choices (coffee and crushed naphthalene). Taste function was assessed by installing two drops of sweet and salty solutions in the anterior two-thirds of the tongue. The United Kingdom Screening Test (UKST) symptom score was used to separate the participants into two groups those with symptoms of peripheral neuropathy (PN) and those without PN.[1] The UKST tool was simple, subjective, and symptom-based instrument, composed of five questions about type, severity and location of symptoms, and a maximum 9 scores. Its cut-off point is > 2. We also performed various simple neurological tests for screening for PN involving tendon reflex, pressure/touch sensation and vibratory sensation.
3. Psychiatric assessment
   a. Assessment of post-traumatic stress disorder (PTSD): We applied the Primary Care PTSD Screen for DSM-5 (PC-PTSD-5), a 5-item screen that is designed for use in primary care settings.[2] The measure begins with an item designed to assess whether the respondent has had any exposure to traumatic events. If a respondent denies exposure, the PC-PTSD-5 is complete with a score of 0. However, if a respondent indicates that they have experienced a traumatic event during the COVID-19 pandemic, the respondent is instructed to respond to five additional yes/no questions about how that trauma exposure has affected them over the past month.
   b. We applied the Mini-Mental State Examination (MMSE) or Folstein test, a 30-point questionnaire to measure cognitive function and to follow the course of cognitive changes in an individual over time. Participants having a score of ≥24 (out of 30) was categorized as having normal cognition. Below this, scores were categorized as having severe (≤9 points), moderate (10–18 points) or mild (19–23 points) cognitive impairment.
   c. We used the Self-Reporting Questionnaire (SRQ-20), a 20-item screening tool, developed by the WHO, to screen for depression and anxiety-related disorders in LMICs. The tool employed a dichotomous answer system with 0 ('no', symptom absent) or 1 ('yes', symptom present). Scores are then summarized to obtain a total score. [3]
   d. We used the Patient Health Questionnaire-9 (PHQ-9) to evaluate the severity of depressive symptoms experienced over the preceding 14 days. The PHQ-9 is a nine-item questionnaire, with each item scored on a scale from 0 (indicating no occurrence) to 3 (almost daily). Threshold scores of 5, 10, 15, and 20 signify mild, moderate, moderately severe, and severe depression, respectively.
   e. We applied the Chalder Fatigue Scale (CFQ-11), an 11-item self-reported questionnaire, to measure the extent and severity of fatigue over the past month. The total score ranges from 0 to 11, with scores ≥4 indicating severe fatigue.[4]
4. Assessment of respiratory and cardiac function
   a. Patients undergo a full clinical assessment using standard clinical procedures.[5]
   b. Dyspnea was evaluated by the modified Medical Research Council (mMRC) scale which consists of five statements that describe almost the entire range of dyspnea from none (Grade 0) to almost complete incapacity (Grade 4). [6]
   c. The Six-minute walk test (6MWT) was performed using the methodology specified by the American Thoracic Society (ATS-2002). [7] The patients were instructed that the objective of this test was to walk as far as possible for 6 minutes. The test was performed in a flat, long corridor, meter-by-meter marked. Heart rate, oxygen saturation, and modified Borg scale assessing subjectively the degree of dyspnea graded from 0 to 10, was recorded at the beginning and the end of the 6MWT. After the 6MWT, the distance covered was calculated.
   d. We performed ECG for all participants at all visits.
   e. Only HS underwent echocardiogram at 5-month and 2-year visits.
5. We applied the Post-COVID-19 Functional Status (PCFS) scale to assess the functional limitations of COVID-19 survivors. This tool identified the full range of functional limitations ranging from no functional

limitation (grade 0) to severe functional limitations (grade 4) due to symptoms, pain, depression or anxiety.[8] We also assessed fatigue by the modified Borg scale, a subjective scale assessing the degree of fatigue from 0 to 10. 0 indicating no fatigue at all, and 10 indicating maximum fatigue.

6. The Medical Research Council (MRC) scale for muscle strength was applied to determine muscle weakness. [5]
7. The nutritional assessment included measuring height, weight, and body mass index (BMI; weight in kilograms divided by square of height in meters)
8. Data about the initial presentation of COVID-19 and the disease course were collected from medical records. If the documentation of symptoms during acute COVID-19 was unavailable in the medical records, patients were asked to recall their symptoms during the acute phase.

S1.2 Outcome measures

1. One or more sequelae symptoms including, fatigue, palpitation, chest pain, breathing difficulty, cough, sputum production, headache, dizziness, red eyes, burning sensation in the mouth, brain fog, sleeping difficulty, loss of appetite, body ache, muscle weakness, Joint pain, any unusual bleeding from any site of the body, nose bleeding, gum bleeding, black/tarry stool, rash on skin, fever, sore throat, runny nose, diarrhea, nausea/vomiting, coughing out of blood, reduced/absent external smell when sniffing, reduced/absent flavor perception when eating, reduced/absent taste (sweet/salt/sour/bitter), presence of smell in absence of stimulus, alteration of the quality of taste, alteration of the quality of smell.
2. Neurological findings including anosmia, absent or altered taste, peripheral neuropathy as evident by the United Kingdom Screening Test score >2, tremor.
3. Psychiatric sequelae as evident by having PTSD (PC-PTSD-5 score >3), depression or anxiety disorder (SRQ-20 score of >5 for male, and > 6 for female respectively out of 20); or cognitive impairment evident by a Mini-Mental State Examination (MMSE) score of <23. We used the PHQ-9 score thresholds of 5, 10, 15, and 20 to signify mild, moderate, moderately severe, and severe depression, respectively. We defined chronic fatigue if the CFQ-11 scores are four or more.[4]
4. Respiratory function abnormalities (mMRC ≥2 scores to quantify residual shortness of breath), tachypnea, abnormal breath sound such as bronchial or diminished breath sound)
5. Cardiovascular findings (hypertension, [9] tachycardia, edema)
6. Poor quality of life (composite outcome) includes fatigue (modified Borg score of ≥1) and the functional limitations of daily activities (the Post-COVID-19 Functional Status score ≥1).
7. Overweight or obesity evident by BMI ≥25
8. Worsening glycaemic control as evident by new requirement of insulin therapy.
9. Hospital readmission and death.
10. Laboratory tests included complete blood count, serum alanine transaminase (ALT), serum creatinine, capillary blood glucose, and urine routine examination.
11. Lung function parameters included forced expiratory volume in one second (FEV1), forced vital capacity (FVC), and the FEV1/FVC ratio. Airflow obstruction was defined as a reduced FEV1/FVC ratio below the 5th percentile of the predicted value. A restrictive spirometry pattern was defined as an FEV1/FVC ratio above the 5th percentile of the predicted value and an FVC below the 5th percentile of the predicted value. Normal lung function was defined as both an FEV1/FVC ratio and an FVC above the 5th percentile of the predicted value.[10,11]
12. ECG abnormality included tachycardia or bradycardia, prolonged PR interval (PR >210ms), short PR interval (PR<119ms), prolonged QTc (QTc >440ms), and ST/T wave changes.
13. Echocardiogram findings included pulmonary hypertension, diastolic relaxation abnormality, ischemic heart disease, cardiomyopathy, pericardial effusion, ejection fraction, and pulmonary arterial systolic pressure.

S1.3 Statistical analysis

We applied a doubly robust propensity score (PS) matching-based approach to standardize the baseline characteristics between HS and NHS. The PS aims to control for measured confounders by attaining balance in baseline characteristics between groups. First, we estimated the PS using a multivariable regression (logistic) model. We used the baseline covariates as a confounder (age, sex, BMI, site, and comorbidity including hypertension, diabetes, ischemic heart disease, chronic liver disease, hypothyroidism, chronic kidney disease, immunocompromised conditions, stroke). We applied a 1:1 PS with a greedy nearest neighbor matching approach

with a caliper width of 0·2.[12] After estimating PS, weights were calculated for each participant as the inverse of the propensity score for HS and as the inverse of (1- propensity score) for NHS.[13] Thus, all baseline characteristics were equally distributed between the cohorts. Standardized mean differences were calculated for the baseline covariates before and after matching. Any covariates with a standardized mean difference of less than 10% were considered well-matched between the cohorts. The proportion of missing explanatory variables in the dataset was unremarkable (1-2%).

We then applied multivariable robust Poisson regression models with robust variance to determine the relative difference in the prevalence of outcome variables between the cohorts at all follow-up visits. Each model was adjusted for all covariates (age, sex, BMI, group, site, and comorbidity) in the PS-matched cohort. The general equation is shown below.

$\ln[E(Y|X)] = \beta 0+\beta 1group+ \beta 2age+ \beta 3sex+ \beta 4bmi+ \beta 5comorbidity+\beta 6site.$

Where, Y=outcome variables (listed below), X=set of covariates

Outcome variables are, any sequelae symptom, fatigue, palpitation, chest pain, breathing difficulty, cough, headache, dizziness, sleeping difficulty, foggy thinking, body ache, muscle weakness, joint pain; any neurologic findings, peripheral neuropathy, anosmia, absent/impaired taste, tremor; psychiatric sequelae, depression/anxiety disorder, post-traumatic stress disorder, cognitive impairment, chronic fatigue syndrome; respiratory findings, mMRC grade 2 or more, bronchial/diminished breath sounds, tachypnea; cardiovascular findings, hypertension, tachycardia, edema; muscle weakness, poor quality of life, required insulin, anemia, high creatinine, proteinuria; normal spirometry findings, restrictive spirometry, obstructive spirometry; any abnormality in ECG, prolonged QTc.

To determine the temporal trend of outcomes among survivors from 9-month to 2-year visits, we applied similarly adjusted (age, sex, BMI, group, site, and comorbidity) generalized estimating equation (GEE) models with log link function and Poisson family. We applied an interaction term between group and time to determine if the relative difference between HS and NHS in outcome measures increases or decreases over time. The GEE model provided a pooled odds ratio (not shown), an estimate of the relative difference between the groups if there was no variation over time. The interaction term informed if the relative difference in the prevalence of outcomes for HS and NHS increased or decreased over time. Similar models were employed for all outcome variables. The general equation is shown below.

$Y \sim X\beta$

Where, Y= outcome variable (as listed above); X= vector of independent variables (age, sex, group, bmi, comorbidity), β = coefficient vector; group coded 1 for hospitalized and 0 for non-hospitalized cohort.

Finally, we employed GEE models to determine the predictors for outcomes (sequelae symptoms, respiratory, and cardiovascular, neurologic, psychiatric outcomes, and chronic fatigue) among all COVID-19 survivors. In the GEE models,' we adjusted age, sex, group, history of diabetes mellitus (DM), history of hypertension, cigarette smoking, site, occupation, and vaccination against COVID-19. The selection of variables was based on biological plausibility and bivariate association in our data. All hypothesis tests were 2-sided. A p-value of <0.05 was considered statistically significant. Although we used robust statistical analysis to control bias, we made many comparisons, and an attempt to formally control the type 1 error across all comparisons was not made; this could be a source of false positives.

References

1. Fateh HR, Madani SP, Heshmat R, Larijani BJJoD, Disorders M. Correlation of Michigan neuropathy screening instrument, United Kingdom screening test and electrodiagnosis for early detection of diabetic peripheral neuropathy. 2015; **15**(1): 1-5.
2. Prins A, Bovin M, Kimerling R, et al. The primary care PTSD screen for DSM-5 (PC-PTSD-5). 2015; **5**: 1-3.
3. van der Westhuizen C, Wyatt G, Williams JK, Stein DJ, Sorsdahl KJIjomh, addiction. Validation of the self reporting questionnaire 20-item (SRQ-20) for use in a low-and middle-income country emergency centre setting. 2016; **14**(1): 37-48.

4. Cleare AJ, Reid S, Chalder T, Hotopf M, Wessely S. Chronic fatigue syndrome. *BMJ clinical evidence* 2015; **2015**.
5. Innes JA, Dover AR, Fairhurst K. Macleod's clinical examination: Elsevier Health Sciences; 2018.
6. Mahler DA, Wells CKJC. Evaluation of clinical methods for rating dyspnea. 1988; **93**(3): 580-6.
7. Med ATSJAJRCC. ATS statement: guidelines for the six-minute walk test. 2002; **166**: 111-7.
8. Klok FA, Boon GJ, Barco S, et al. The Post-COVID-19 Functional Status scale: a tool to measure functional status over time after COVID-19. 2020; **56**(1).
9. Zhou B, Carrillo-Larco RM, Danaei G, et al. Worldwide trends in hypertension prevalence and progress in treatment and control from 1990 to 2019: a pooled analysis of 1201 population-representative studies with 104 million participants. 2021; **398**(10304): 957-80.
10. Jankowich M, Elston B, Liu Q, et al. Restrictive Spirometry Pattern, Cardiac Structure and Function, and Incident Heart Failure in African Americans. The Jackson Heart Study. *Annals of the American Thoracic Society* 2018; **15**(10): 1186-96.
11. Sylvester KP, Clayton N, Cliff I, et al. ARTP statement on pulmonary function testing 2020. *BMJ open respiratory research* 2020; **7**(1).
12. Austin PCJMbr. An introduction to propensity score methods for reducing the effects of confounding in observational studies. 2011; **46**(3): 399-424.
13. Chesnaye NC, Stel VS, Tripepi G, et al. An introduction to inverse probability of treatment weighting in observational research. 2022; **15**(1): 14-20.

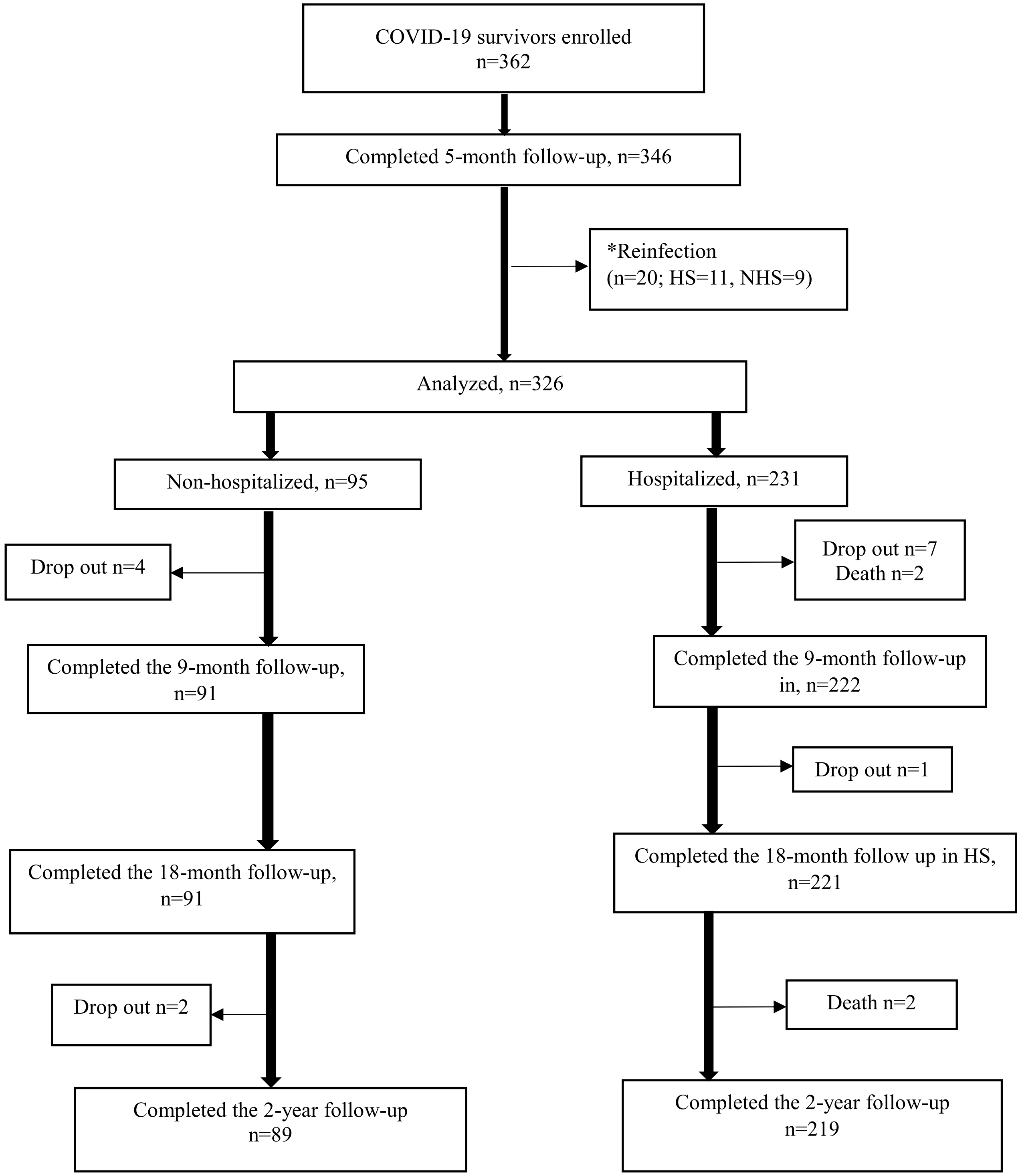

Figure S1: Flow diagram of the study participants. HS: hospitalized survivors; NHS: non-hospitalized survivors.

Table S1: Characteristics of the hospitalized and non-hospitalized COVID-19 survivors after propensity score matching

	Original Sample			Matched Sample		
	**Hospitalized Survivors (n=231)**	**Non-hospitalized Survivors (n=95)**	**SMD**	**Hospitalized Survivors (n=231)**	**Non-hospitalized Survivors (n=95)**	**SMD**
Age in years	52.762	42.358	10.404	52.762	52.978	-0.216
Sex	0.641	0.579	0.062	0.641	0.706	-0.065
BMI	27.720	27.580	0.140	27.720	27.27	0.450
Comorbidity	0.758	0.505	0.253	0.758	0.736	0.022
Site	1.424	1.242	0.182	1.424	1.385	0.039

SMD=Standardized mean difference; BMI, body mass index

Table S2: Sequelae-symptoms among non-hospitalized and hospitalized COVID-19

	5-month follow-up							9-month follow-up							18-month follow-up							2-year follow-up							**Change over time in NHS	**Change over time in HS
	Total (326)		NHS (95)		HS (231)			Total (313		NHS (91)		HS (222)			Total (312		NHS (91		HS (221)			Total (30		NHS (89		HS (219)				
	n	%	n	%	n	%	RR (95% CI)	n	%	n	%	n	%	RR (95% CI)	n	%	n	%	n	%	RR (95% CI)	n	%	n	%	n	%	RR (95% CI)		
**Most Common: >10%**																														
Fatigue	164	50	42	44	122	53	1.1 (0.8, 1.5)	172	55	40	44	132	59	1.3 (1.1, 1.6)	160	51	44	48	116	52	1.1 (0.8, 1.4)	##	38	26	29	92	42	1.3 (0.8, 1.8)	0.219	0.067
Sleeping difficulty	106	33	23	24	83	36	1.2 (0.8, 1.8)	97	31	22	24	75	34	1.1 (0.7, 1.7)	98	31	24	26	74	33	1.1 (0.8, 1.7)	75	24	14	16	61	28	1.4 (1.1, 1.9)	0.297	0.128
Joint pain	106	33	21	22	85	37	1.5 (1.2, 1.9)	128	41	34	37	94	42	1.1 (0.8, 1.5)	114	37	21	23	93	42	1.6 (1.1, 2.5)	##	36	20	22	89	41	1.3 (1.0, 1.7)	0.581	0.651
Weight gain	102	31	24	25	78	34	1.4 (0.9, 2.1)	119	38	32	35	87	39	1.1 (0.8, 1.6)	106	34	32	35	74	33	1.0 (0.7, 1.4)	##	34	34	38	71	32	0.9 (0.6, 1.3)	0.085	0.557
Headache	94	29	23	24	71	31	1.4 (1.1, 1.8)	105	34	34	37	71	32	0.9 (0.6, 1.3)	88	28	29	32	59	27	1.0 (0.6, 1.4)	56	18	11	12	45	21	1.6 (1.1, 2.4)	0.144	0.021
Body ache	75	23	19	20	56	24	1.1 (0.7, 1.8)	85	27	24	26	61	27	1.0 (0.7, 1.5)	89	29	26	29	63	29	1.0 (0.7, 1.5)	58	19	15	17	43	20	1.1 (0.6, 1.8)	0.803	0.263
Weight loss	68	21	19	20	49	21	1.0 (0.6, 1.7)	55	18	19	21	36	16	0.7 (0.4, 1.1)	79	25	21	23	58	26	1.1 (0.7, 1.8)	74	24	18	20	56	26	1.2 (0.7, 1.9)	0.905	0.049
Dizziness	63	19	13	14	50	22	1.2 (0.7, 2.3)	62	20	19	21	43	19	0.9 (0.6, 1.5)	54	17	10	11	44	20	1.5 (1.1, 2.2)	37	12	4	4	33	15	2.5 (1.5, 4.3)	0.040	0.119
Palpitation	63	19	16	17	47	20	1.0 (0.6, 1.7)	48	15	14	15	34	15	1.0 (0.5, 1.9)	44	14	10	11	34	15	1.1 (0.6, 2.3)	35	11	4	4	31	14	2.8 (1.0, 7.8)	0.020	0.129
Muscle weakness	58	18	13	14	45	19	1.2 (0.6, 2.2)	66	21	13	14	53	24	1.6 (1.2, 2.3)	86	28	22	24	64	29	1.1 (0.7, 1.7)	69	22	11	12	58	26	1.6 (1.1, 2.2)	0.728	0.101
Loss of appetite	61	19	15	16	46	20	1.1 (0.6, 1.9)	56	18	9	10	47	21	1.9 (1.3, 2.7)	50	16	9	10	41	19	1.5 (0.7, 3.0)	27	9	4	4	23	11	1.8 (0.7, 5.1)	0.010	0.012
Blurred vision	56	17	13	14	43	19	1.4 (0.7, 2.6)	67	21	19	21	48	22	1.0 (0.6, 1.7)	51	16	17	19	34	15	0.9 (0.5, 1.5)	42	14	11	12	31	14	1.0 (0.5, 1.9)	0.932	0.140
Brain fog	54	17	13	14	41	18	1.1 (0.6, 2.0)	52	17	11	12	41	18	1.4 (0.7, 2.6)	40	13	11	12	29	13	1.0 (0.5, 2.1)	36	12	7	8	29	13	1.6 (1.0, 2.4)	0.312	0.115
Cough	41	13	7	7	34	15	1.6 (1.1, 2.5)	90	29	20	22	70	32	1.4 (1.1, 1.9)	77	25	20	22	57	26	1.1 (0.7, 1.8)	45	15	9	10	36	16	1.3 (0.6, 2.7)	0.653	0.897
Hearing loss	38	12	6	6	32	14	1.6 (0.6, 3.7)	30	10	10	11	20	9	0.9 (0.5, 1.9)	29	9	7	8	22	10	1.2 (0.6, 2.9)	22	7	3	3	19	9	1.4 (0.4, 5.3)	0.333	0.079
Chest pain	38	12	7	7	31	13	1.6 (1.0, 2.4)	54	17	15	16	39	18	1.0 (0.6, 1.8)	36	12	8	9	28	13	1.2 (0.5, 2.5)	27	9	7	8	20	9	1.0 (0.4, 2.4)	0.730	0.073
Loss of smell sense	35	11	10	11	25	11	0.8 (0.5, 1.2)	22	7	5	5	17	8	1.0 (0.6, 1.8)	23	7	8	9	15	7	0.8 (0.3, 1.9)	12	4	5	6	7	3	0.5 (0.1, 1.6)	0.394	0.003
Runny nose	34	10	10	11	24	10	1.1 (0.7, 1.6)	46	15	19	21	27	12	0.7 (0.4, 1.2)	36	12	16	18	20	9	0.6 (0.3, 1.1)	21	7	3	3	18	8	2.0 (0.6, 6.7)	0.188	0.379
Fever	38	12	5	5	33	14	2.7 (1.1, 6.6)	78	25	22	24	56	25	1.0 (0.7, 1.6)	81	26	24	26	57	26	1.0 (0.7, 1.5)	37	12	8	9	29	13	1.9 (1.3, 3.0)	0.460	0.909
Burning sensation in the mouth	32	10	8	8	24	10	1.3 (0.6, 3.2)	43	14	11	12	32	14	1.0 (0.5, 2.0)	43	14	13	14	30	14	1.0 (0.5, 1.9)	13	4	3	3	10	5	1.0 (0.3, 3.4)	0.471	0.076
Breathing difficulty	31	10	7	7	24	10	1.6 (1.1, 2.6)	31	10	7	8	24	11	1.4 (0.6, 3.1)	43	14	7	8	36	16	1.6 (0.7, 3.5)	41	13	6	7	35	16	1.5 (0.6, 3.5)	0.955	0.035
**Less Common: <10%**																														
Rash	29	9	6	6	23	10	1.7 (0.7, 4.2)	31	10	11	12	20	9	0.8 (0.4, 1.9)	22	7	7	8	15	8	0.9 (0.3, 2.3)	16	5	4	4	12	5	1.0 (0.3, 3.7)	0.619	0.083
Sore throat	25	8	10	11	15	6	0.7 (0.3, 1.5)	3	1	1	1	2	1	1.0 (0.3, 3.4)	0	0	0	0	0	0	NA	1	0.3	0	0	1	0.5	NA	0.012	<0.001
Absence of flavour when eating	23	7	4	4	19	8	1.3 (0.4, 3.9)	10	3	3	3	7	3	1.0 (0.3, 4.3)	17	5	5	5	12	5	1.0 (0.4, 2.8)	6	2	2	2	4	2	0.9 (0.1, 6.2)	0.672	0.009
Absent smell	23	7	7	7	16	7	0.9 (0.4, 2.2)	15	5	4	4	11	5	1.1 (0.4, 3.3)	15	5	5	5	10	5	0.9 (0.3, 2.7)	7	2	2	2	5	2	0.9 (0.2, 4.6)	0.200	0.032
Sputum production	24	7	5	5	19	8	1.4 (0.5, 3.7)	44	14	10	11	34	15	1.3 (0.6, 2.7)	30	10	5	5	25	11	1.6 (0.7, 4.1)	21	7	6	7	15	7	1.1 (0.4, 3.2)	0.802	0.550
Diarrhea	20	6	7	7	13	6	0.7 (0.3, 1.8)	74	24	20	22	54	24	1.1 (0.7, 1.7)	71	23	23	25	48	22	0.9 (0.6, 1.5)	39	13	8	9	31	14	1.6 (0.7, 3.3)	0.482	0.023
Nausea	17	5	6	6	11	5	0.8 (0.3, 2.0)	37	12	10	11	27	12	1.1 (0.5, 2.2)	30	10	8	9	22	10	1.1 (0.5, 2.4)	21	7	5	6	16	7	1.1 (0.4, 2.8)	0.729	0.440
Red eyes	10	3	1	1	9	4	2.3 (0.4, 14.3)	10	3	2	2	8	4	1.5 (0.2, 10.1)	13	4	4	4	9	4	0.9 (0.3, 2.9)	3	1	0	0	3	1	NA	0.951	0.232
Altered taste sensation	13	4	1	1	12	5	3.4 (0.4, 29.7)	7	2	0	0	7	3	NA	9	3	2	2	7	3	1.1 (0.3, 4.3)	2	1	0	0	2	1	NA	0.830	0.016
Absent taste sensation	13	4	2	2	11	5	1.3 (0.3, 6.5)	8	3	2	2	6	3	1.0 (0.2, 6.1)	8	3	2	2	6	3	1.0 (0.1, 6.5)	4	1	0	0	4	2	NA	0.160	0.008
Altered smell	10	3	4	4	6	3	0.7 (0.2, 2.4)	5	2	1	1	4	2	1.1 (0.1, 12.6)	4	1	2	2	2	1	0.5 (0.1, 3.2)	1	0.3	1	1	0	0	NA	0.303	0.022
Altered smell	9	3	1	1	8	3	1.7 (0.2, 13.6)	5	2	1	1	4	2	1.0 (0.1, 9.8)	5	2	1	1	4	2	1.1 (0.1, 8.5)	2	1	1	1	1	0.5	0.3 (0.1, 6.3)	0.822	0.031
Black or tarry stool	7	2	4	4	3	1	0.3 (0.1, 1.3)	7	2	2	2	5	2	1.0 (0.2, 5.1)	11	4	4	4	7	3	0.8 (0.2, 2.9)	3	1	2	2	1	0.5	0.2 (0.1, 2.2)	0.674	0.753
Unusual bleeding	6	2	3	3	3	1	0.4 (0.1, 2.4)	10	3	2	2	8	4	1.1 (0.2, 6.4)	15	5	6	7	9	4	0.7 (0.2, 1.8)	9	3	2	2	7	3	1.5 (0.3, 7.1)	0.728	0.184
Nose bleeding	1	0.3	0	0	1	0.4	NA	3	1	0	0	3	1	NA	2	1	0	0	2	1	NA	2	1	0	0	2	1	NA	NA	0.582
Coughing out blood	1	0.3	1	1	0	0	NA	19	6	6	7	13	6	1.0 (0.3, 2.8)	19	6	4	4	15	7	1.4 (0.5, 4.4)	7	2	2	2	5	2	1.0 (0.2, 5.6)	0.685	0.144
Gum bleeding	1	0.3	0	0	1	0.4	NA	4	1	1	1	3	1	1.2 (0.1, 11.5)	3	1	2	2	1	0.5	0.2 (0.1, 1.9)	5	2	1	1	4	2	1.6 (0.1, 30.1)	0.219	0.258

Data are n (%) or median (IQR). RR: risk ratio; CI: confidence interval. NHS: non hospitalized survivors; HS: hospitalized survivors. NA: not applicable.

*All estimates are adjusted for age, sex, body mass index, any comorbidity, group (hospitalized/non-hospitalized), and site (BSMMU/icddrb).

**P value is for the interaction between the follow-up visit and group. P value is derived from the generalized estimating equation (GEE) models, indicating the trend of changing characteristics over time.

The pooled risk ratios from GEE models are not shown.

Table S3: Depressive symptoms among non-hospitalized and hospitalized COVID-19 survivors

	**9-month follow-up visit**				**18-month follow-up visit**				**24-month follow-up visit**				****Change over time in NHS**	****Change over time in HS**
	**Total (327)**	**NHS (97)**	**HS (230)**	**RR (95% CI)**	**Total (325)**	**NHS (97**	**HS (228)**	**RR (95% CI)**	**Total (325)**	**NHS (96)**	**HS (229)**	**RR (95% CI)**		
**Depressive symptoms**	**144 (44)**	**38 (39)**	**106 (46)**	**1·03 (0.77, 1.40)**	**117 (36)**	**28 (29)**	**89 (39)**	**1·23 (0.84, 1.82)**	**105 (32)**	**21 (22)**	**84 (37)**	**1.62 (1.04, 2.54)**	**0.024**	**0.147**
No depression	74 (23)	29 (30)	45 (20)	0·74 (0.48, 1.16)	93 (29)	39 (40)	54 (24)	0.64 (0.44, 0.91)	80 (25)	34 (35)	46 (20)	0·74 (0.50, 1.10)	0.982	0.734
Mild depression	185 (57)	52 (54)	133 (58)	1.05 (0.84, 1.32)	199 (61)	50 (52)	149 (65)	1.26 (1.02, 1.57)	216 (66)	58 (60)	158 (69)	1.12 (0.92, 1.36)	0.652	0.659
Moderate to severe depression	68 (21)	16 (16)	52 (23)	1.28 (0.77, 2.13)	33 (10)	8 (8)	25 (11)	1.10 (0.49, 2.49)	29 (9)	4 (4)	25 (11)	2.77 (1.00, 7.67)	0.078	0.004

Data are n (%) or median (IQR). RR: adjusted risk ratio; CI: confidence interval. NHS: non-hospitalized survivors; HS: hospitalized survivors. NA: not applicable. PHQ9: Patient Health Questionnaire-9 (PHQ-9)

No depression: PHQ9 score=0; mild depression: PHQ9 score 1-9; moderate to severe depression: PHQ9 score 10-27.

*All estimates are adjusted for age, sex, body mass index, any comorbidity, group (hospitalized/non-hospitalized), and site (BSMMU/icddrb).

**P value is for the interaction between the follow-up visit and group. P value is derived from the generalized estimating equation (GEE) models, indicating the trend of changing characteristics over time.

The pooled risk ratios from GEE models are not shown.

Table S4: Causes of hospitalization in non-hospitalized and hospitalized COVID-19 survivors

	5-month visit			9-month visit			18-month visit			2-year visit		
	Total (326)	NHS (95)	HS (231)	Total (313)	NHS (91)	HS (222)	Total (312)	NHS (91)	HS (221)	Total (308)	NHS (89)	HS (219)
**Required hospitalizations**	**6 (2)**	**1 (1)**	**5 (2)**	**13 (4)**	**4 (4)**	**9 (4)**	**16 (5)**	**2 (2)**	**14 (6)**	**10 (3)**	**3 (3)**	**7 (3)**
All causes of hospitalization												
Cardiac cause	2 (1)	0 (0)	2 (1)	1 (0.3)	0 (0)	1 (0.5)	5 (2)	0 (0)	5 (2)	2 (1)	0 (0)	2 (1)
Infections	3 (1)	1 (1)	2 (1)	5 (2)	3 (3)	2 (1)	2 (1)	0 (0)	2 (1)	5 (2)	3 (3)	2 (1)
Stroke	0 (0)	0 (0)	0 (0)	2 (1)	0 (0)	2 (1)	0 (0)	0 (0)	0 (0)	0 (0)	0 (0)	0 (0)
Other cause	1 (0.3)	0 (0)	1 (0.4)	5 (2)	1 (1)	4 (2)	9 (3)	2 (2)	7 (3)	3 (1)	0 (0)	3 (1)

Data are n (%). NHS: non-hospitalized survivors; HS: hospitalized survivors.

Table S5: Incidence of new sequelae-symptom and clinical and laboratory outcomes among COVID-19 survivors.

	**Total COVID-19 survivors (326)**		**Non-hospitalized survivors (95)**		**Hospitalized survivors (231)**		**Adjusted risk ratio**	
	Number	Cases per 1000 person-year	Number	Cases per 1000 person-year	Number	Cases per 1000 person-year	Estimates	*P*-value
**Any symptom-sequelae**	87	137.39	34	184.78	53	117.97	0.90 (0.59– 1.36)	0.609
Joint pain	144	227.39	40	217.39	104	231.49	1.05 (0.80– 1.39)	0.705
Body ache	104	164.23	30	163.04	74	164.72	1.02 (0.71– 1.45)	0.923
Fatigue	109	172.13	29	157.61	80	178.07	1.27 (0.85– 1.89)	0.243
Headache	118	186.34	35	190.22	83	184.75	1.04 (0.73– 1.47)	0.835
Dizziness	91	143.70	25	135.87	66	146.91	1.01 (0.67– 1.51)	0.973
Sleeping difficulty	112	176.87	32	173.91	80	178.07	1.04 (0.73– 1.47)	0.826
Muscle weakness	108	170.55	29	157.61	79	175.85	1.04 (0.72– 1.49)	0.841
Brain fog	72	113.69	16	86.96	56	124.65	1.68 (0.99– 2.86)	0.053
Palpitation	71	112.12	16	86.96	55	122.43	1.67 (1.01– 2.80)	0.047
Cough	143	225.82	34	184.78	109	242.63	1.43 (1.03– 1.99)	0.034
Chest pain	67	105.80	22	119.57	45	100.17	0.82 (0.52– 1.29)	0.393
**Clinical outcomes**								
Any neurologic outcomes	127	200.55	35	190.22	92	204.78	1.05 (0.76– 1.45)	0.774

Peripheral neuropathy	111	175.29	32	173.91	79	175.85	1.00 (0.72– 1.39)	0.990
Any cardiovascular outcomes	133	210.03	29	157.61	104	231.49	1.44 (1.03– 2.02)	0.034
Hypertension	113	178.44	24	130.43	89	198.11	1.46 (1.00– 2.12)	0.047
Any respiratory outcomes	99	156.34	37	201.09	62	138.01	0.68 (0.49– 0.95)	0.022
MMRC grade or 2 or more	91	143.70	30	163.04	61	135.78	0.84 (0.57– 1.22)	0.352
Depression/anxiety disorder	91	143.70	22	119.56	69	153.59	1.48 (0.95– 2.29)	0.079
Post-traumatic stress disorder	20	31.58	5	27.17	15	33.39	1.13 (0.39– 3.30)	0.824
Cognitive impairment	27	42.64	9	48.91	18	40.07	0.95 (0.34– 2.68)	0.927
Chronic fatigue	63/313	101.04	19/91	104.97	44/222	99.44	0.97 (0.57– 1.64)	0.902
Muscle weakness	40	63.16	13	70.65	27	60.10	0.64 (0.33– 1.24)	0.226
Required insulin	40	63.16	8	43.48	32	71.23	1.05 (0.51– 2.17)	0.896
Diabetes Mellitus	4	6.32	1	5.43	3	6.68	0.78 (0.08– 7.76)	0.840
Hospitalization	50	78.96	15	81.52	35	77.91	0.74 (0.42– 1.32)	0.341
Overweight/obese	29	45.79	7	38.04	22	48.97	1.39 (0.58– 3.31)	0.460
**Laboratory outcomes**								
Any abnormality in ECG	81	127.91	23	125.00	58	129.10	1.06 (0.68– 1.66)	0.790

Restrictive pattern on spirometry	85	134.23	22	119.56	63	140.23	1.18 (0.77– 1.83)	0.442
Anemia⸸	72	113.69	19	103.26	53	117.97	1.29 (0.78– 2.14)	0.318
High creatinine†	22	34.74	5	27.17	17	37.84	1.03 (0.37– 2.82)	0.959
High ALT£	103	162.65	31	168.48	72	160.27	1.06 (0.70– 1.58)	0.794
Proteinuria€	31	48.95	7	38.04	24	53.42	1.23 (0.56– 2.71)	0.601

*All estimates are adjusted for age, sex, body mass index, any comorbidity, group (non-hospitalized/hospitalized), and site (icddr,b/BSMMU).
⸸ Hemoglobin (g/dl) <13 for male, and <12 for female; † high creatinine >106 mmol/L; £ALT (alanine transaminase) >40 U/L; €one plus or more protein in urine.

Table S6: Risk factors for symptom-sequelae, respiratory cardiovascular, neurologic, psychiatric outcomes, and chronic fatigue syndrome among COVID-19 survivors.

	**Sequelae-symptom∫**	**Respiratory outcomes†**	**Cardiovascular outcomes£**	**Neurologic outcomes¥**	**Psychiatric outcomesð**	**Chronic fatigue€**
	aRR (95% CI)	aRR (95% CI)	aRR (95% CI)	aRR (95% CI)	aRR (95%CI)	aRR (95%CI)
Age Group⸸						
40-60 years	1.24 (0.99– 1.54)	1.51 (1.05– 2.19)	1.41 (0.98– 2.02)	1.67 (1.15– 2.43)	1.03 (0.73– 1.45)	1.34 (0.91– 1.97)
>60 years	1.22 (0.90– 1.65)	1.95 (1.24– 3.05)	2.23 (1.43– 3.48)	1.98 (1.26– 3.13)	1.10 (0.70– 1.73)	1.34 (0.81– 2.23)
Female⸸	1.24 (0.99– 1.54)	1.56 (1.12– 2.17)	1.29 (0.93– 1.78)	1.73 (1.25– 2.38)	2.25 (1.60– 3.17)	1.78 (1.23– 2.58)
Hospitalized survivors⸸	1.05 (0.86– 1.28)	1.04 (0.77– 1.40)	1.17 (0.86– 1.59)	1.25 (0.91– 1.70)	1.02 (0.75– 1.38)	1.09 (0.77– 1.54)
Cigarette smoking	1.13 (0.88– 1.45)	1.11 (0.74– 1.67)	0.98 (0.66– 1.44)	1.11 (0.74– 1.67)	1.56 (1.02– 2.37)	1.38 (0.89– 2.14)
BSMMU site	1.08 (0.91– 1.28)	0.99 (0.76– 1.28)	1.05 (0.81– 1.36)	1.02 (0.78– 1.31)	0.97 (0.74– 1.27)	1.14 (0.85– 1.52)
Occupation⸸						
Unemployed	1.00 (0.79– 1.27)	1.20 (0.85– 1.70)	0.78 (0.54– 1.12)	1.01 (0.72– 1.42)	1.11 (0.79– 1.55)	1.00 (0.68– 1.48)
Retired	1.16 (0.86– 1.57)	1.30 (0.85– 1.99)	0.96 (0.64– 1.44)	1.15 (0.75– 1.76)	1.16 (0.72– 1.89)	1.35 (0.82– 2.23)
≥2 doses of COVID-19 vaccines⸸	0.94 (0.83– 1.08)	0.76 (0.63– 0.91)	0.83 (0.68– 1.02)	0.85 (0.66– 1.09)	0.78 (0.66– 0.92)	0.92 (0.70– 1.20)
Diabetes	1.01 (0.84– 1.22)	1.31 (0.99– 1.71)	1.34 (1.02– 1.74)	1.31 (1.01– 1.71)	1.33 (0.99– 1.77)	1.04 (0.76– 1.42)

aRR = Adjusted Risk ratio, CI = confidence interval.

∫Reference group: age <40 years; male; non-hospitalized; employed; no vaccine or one dose of vaccine

Sequelae-symptom⸸ = any symptom listed in Table 2 and S2 in Online supplementary document; Respiratory outcomes† = mMRC (modified Medical Research Council) dyspnea grade ≥2, bronchial or diminished breath sound, tachypnea, hypoxemia; Cardiovascular outcomes£ = hypertension, tachycardia, edema; Neurologic outcomes¥ = peripheral neuropathy, anosmia, absent/impaired taste, tremor; Psychiatric outcomesð = Depression/anxiety disorder, Post-traumatic stress disorder cognitive impairment; Chronic fatigue€ = the Chalder Fatigue Scale score ≥4.

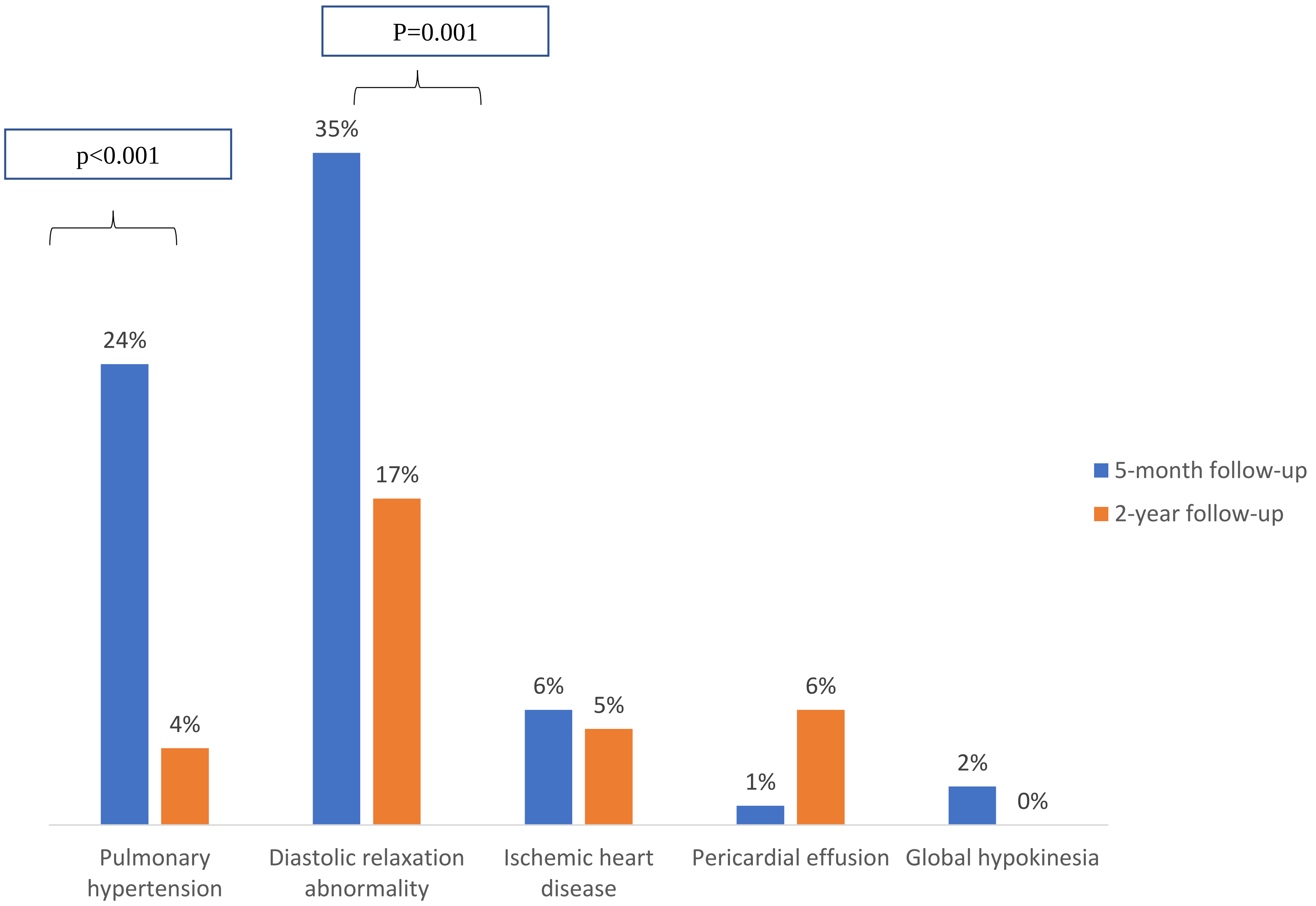

Figure S2: Echocardiogram findings among hospitalized COVID-19 survivors at 5 months and 2 year time points.
